# Kinocardiography Derived from Ballistocardiography and Seismocardiography Shows High Repeatability in Healthy Subjects

**DOI:** 10.3390/s21030815

**Published:** 2021-01-26

**Authors:** Amin Hossein, Jérémy Rabineau, Damien Gorlier, Jose Ignacio Juarez Del Rio, Philippe van de Borne, Pierre-François Migeotte, Antoine Nonclercq

**Affiliations:** 1 LPHYS, Université Libre de Bruxelles, 1050 Bruxelles, Belgium; jeremy.rabineau@ulb.be (J.R.); Damien.gorlier@ulb.ac.be (D.G.); pierre-francois.migeotte@ulb.ac.be (P.-F.M.); 2 BEAMS, Université Libre de Bruxelles, 1050 Bruxelles, Belgium; anoncler@ulb.ac.be; 3 Department of Cardiology, Erasme Hospital, Université Libre de Bruxelles, 1050 Bruxelles, Belgium; joseignacio.juarez@salud.madrid.org (J.I.J.D.R.); philippe.van.de.borne@erasme.ulb.ac.be (P.v.d.B.)

**Keywords:** cardiac contractility, cardiac kinetic energy, test–retest, repeatability, reliability, ballistocardiography, seismocardiography, kinocardiography, cardiac monitoring

## Abstract

Recent years have witnessed an upsurge in the usage of ballistocardiography (*BCG*) and seismocardiography (*SCG*) to record myocardial function both in normal and pathological populations. Kinocardiography (KCG) combines these techniques by measuring 12 degrees-of-freedom of body motion produced by myocardial contraction and blood flow through the cardiac chambers and major vessels. The integral of kinetic energy (*iK*) obtained from the linear and rotational *SCG*/*BCG* signals, and automatically computed over the cardiac cycle, is used as a marker of cardiac mechanical function. The present work systematically evaluated the test–retest (TRT) reliability of KCG *iK* derived from *BCG*/*SCG* signals in the short term (<15 min) and long term (3–6 h) on 60 healthy volunteers. Additionally, we investigated the difference of repeatability with different body positions. First, we found high short-term TRT reliability for KCG metrics derived from *SCG* and *BCG* recordings. Exceptions to this finding were limited to metrics computed in left lateral decubitus position where the TRT reliability was moderate-to-high. Second, we found low-to-moderate long-term TRT reliability for KCG metrics as expected and confirmed by blood pressure measurements. In summary, KCG parameters derived from *BCG*/*SCG* signals show high repeatability and should be further investigated to confirm their use for cardiac condition longitudinal monitoring.

## 1. Introduction

Telemonitoring is a relatively new but quickly developing area in medicine. This is particularly the case with regard to the monitoring of cardiac activity, where detecting the heart’s weakness at the onset of a cardiac incident would allow us to act early and avoid further irreversible damage [1]. Furthermore, recent global health challenges, such as the COVID-19 pandemic, have shed new light on the use of teleconsultation and telemedicine. Indeed, since the start of this epidemic, cardiovascular disease (CVD) has been quickly identified as an important factor in co-morbidity [2]. COVID-19 interacts with the cardiovascular system at several levels, increasing morbidity in patients with underlying CVD and causing myocardial damage and dysfunction ([3], p. 19). Additionally, during the pandemic, some chronic cardiac patients have avoided going to the hospital or to their doctor due to fear of COVID-19 contagion ([4], p. 19). In these conditions, portable tools allowing us to regularly and reliably measure the cardiac chronotropic linked to heart rate and inotropic linked to myocardial contractility states are needed.

Due to new technological improvements, namely, in the field of accelerometers and gyroscopes sensors, recent years have witnessed an upsurge in the use of ballistocardiography (*BCG*) and seismocardiography (*SCG*) to record myocardial function both in normal and pathological populations [5,6]. *BCG* and *SCG* are techniques that can be used to assess the inotropic state based on the measurement of body movements induced by cardiac contraction and blood flow in the cardiac chambers and major vessels [7,8]. The relationship between cardiac contractility in the broad sense and the ballistic signals was identified, and extensively studied, by Starr in the twentieth century [7].

More recently, these techniques have been used in areas such as atrial fibrillation detection [9], heart failure monitoring [10] and cardiorespiratory fitness [11].

Kinocardiography (KCG) is a subject-specific calibrated combination of linear and rotational *SCG* and *BCG* techniques. KCG is based on measures of 6 degrees-of-freedom (DOF)—from three-dimensional (3D) linear and 3D angular motion—recorded from sensors attached to the sternum (*SCG*) and 6-DOF—3D linear and 3D angular—from whole-body motion recorded at the lumbar area (*BCG*). The main contribution of KCG is to provide comprehensive scalar metrics, introduced as the time integral of kinetic energy (*iK*) over the cardiac cycle. These metrics provide a measure of the intensity of cardiac mechanical activity. They were introduced and discussed in a previous randomized, double-blind and placebo-controlled validation study [12]. In the latter, increasing levels of dobutamine were infused to healthy subjects inducing changes in cardiac contractility and heart rate. Differences in KCG metrics were associated with differences in stroke volume (SV) and cardiac output (CO) [12]. These metrics have been shown to be computable based on *BCG* and *SCG* signals acquired using different sensors at either 1 kHz or 50 Hz indifferently [13]. In other studies, differences in KCG metrics have also been associated with an increase during voluntary apnea [14], an increase during simulated obstructive apnea [15], an increase during sympathetic activation [16] and a decrease during a deconditioning that occurred during long-duration head-down tilt bed rest [17]. Establishing the reliability of these measures is crucial to the continued investigation of such interindividual and group-based differences. *BCG* repeatability was studied with contradictory results [18,19,20]. Recently, short- and long-term (one hour and one day, respectively) systolic time interval repeatability derived from *SCG* signals was studied by Vahid Zakeri et al. [21]. To our knowledge, the repeatability of the *SCG* amplitude signal on a single axis or a combination of several axes was not studied. No prior study has quantified the test–retest reliability of KCG (combining *SCG* and *BCG*).

**Figure 1:**
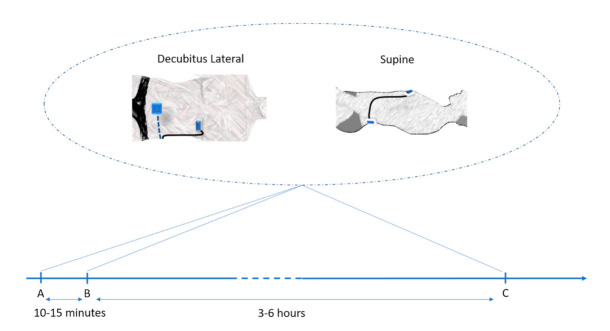
Study protocol. Data were acquired in 2 configurations—left lateral decubitus (LD) and supine (Sup)—and at 3 points in time—Point A, Point B after 10 to 15 min and Point C after 3 to 6 h resulting in a total of 6 data recordings.

In the present study, we investigated the TRT reliability of KCG metrics. Specifically, we used KCG metrics to measure resting-state activity in a group of 60 healthy participants at three different points in time, to assess intersession (>3 h apart) and intrasession (<15 min apart) reliability. At each time point, two configurations were measured at different body positions (Figure 1).

We then explored the reliability and consistency of KCG metrics in each configuration. We computed the following: (1) the TRT reliability of correlations between pairs of KCG metrics using intraclass correlation (ICC) intra- and intersession; (2) the coefficient of variation (*CV*) intrasession; (3) the influence of body position on KCG parameters.

## 2. Materials and Methods

### 2.1. Protocols and Participants

In total, 60 non-smoking healthy volunteers with no history of cardiac disease and a BMI between 20 and 25 kg/m^2^ were recruited. Participants had a mean age of 24.4 years (±1.5), a BMI of 22.2 kg/m^2^ (±2.4) and 30 (50%) were females. None of the participants took any drugs or medications. The study protocol complied with the Declaration of Helsinki, and was approved by the local Ethics Committee (Hôpital Erasme—CCB: B406201630013). The prototype device used in this clinical trial was authorized by the Belgian Federal Agency for Medicine and Health Products (FAMHP). Written informed consent was obtained from each participant prior to the experimental testing procedure.

The timeline of the experimentation is shown in Figure 1. In the morning, after being equipped with the kinocardiograph described in Section 2.3, volunteers were instructed to lie in supine position on a bed for 5 min for stabilization. A blood-pressure measure was performed (Omron, EVOLV, HEM-7600T-E, Japan). A familiarization KCG recording was then acquired for 300 s. Volunteers were then instructed to lie in left lateral decubitus position. After 5 min of stabilization, KCG of the same duration was acquired, called lateral decubitus (LD). Volunteers were then instructed to lie in supine position and, after 5 min of stabilization, another acquisition was performed, called supine (Sup). Volunteers were then completely de-instrumented. After 5 min, they were re-instrumented, and recordings in the 2 configurations described above were performed again. The volunteers were de-instrumented and asked to come back after 3 to 6 h. Blood pressure was then measured again, and the recordings in the 2 configurations described above were performed again. In this study, only intraday repeatability was considered since we hypothesized that short-term intraday repeatability likely deals only or mainly with technical or technological repeatability, which is what we investigate here, while interday repeatability will likely deal with physiological stability of the parameters extracted from KCG.

#### Breathing Protocol

The influence of breathing on accelerometric records such as the *BCG* is well known [22]. Records are standardized with an Imposed and Controlled Breathing (ICB) protocol, which proved useful when comparing intra- and inter-subject records [23]. This ICB protocol consisted of 4 × 10 repetitions of a fixed length breathing pattern. The participants were instructed via a sound indicating the breathing pattern to follow and had to successively perform 10 repetitions of 4, 6, 8 and 10 s breathing cycles. In this study, only data from the 10 s breathing cycle protocol (i.e., the longest record that can be achieved within a constant breathing cycle) are presented.

### 2.2. Portable Acquisition Device

The Kinocardiograph is a wearable device with two detectors, one of which is placed over the lumbar region close to the subject’s center of mass and the other on the chest. Each detector contains a microelectromechanical systems (MEMS) accelerometer and gyroscope sensor (LSM6DSL, STMicroelectronics) and is attached to the body with standard sticky gel electrodes—the lumbar detector is further secured in place with an elastic band. The acceleration and angular rates of the sensor were set to ±2 g and ±250 dps, respectively with a resolution of 0.061 mg/LSB and 4.375 mdps/LSB and an RMS noise of 80  μgHz and 4  mdpsHz with an output bandwidth of 416 Hz. The Kinocardiograph is controlled with a smartphone or a tablet connected via Bluetooth and collects a two-lead electrocardiogram (ECG) at 200 Hz (ADS1292R, AD Instruments) together with 3-DOF linear (LIN) accelerations and 3-DOF rotational (ROT) angular velocities from the chest (*SCG*) and the lumbar region (*BCG*). In brief, a total of 12-DOF linear acceleration and angular velocity signals were recorded at 50 Hz and filtered with a 25 Hz hardware low-pass filter. A 4th order Butterworth IIR filter with pass bands 0.5–60 Hz was applied to the ECG signals.

The standard nomenclature [24] was used: for *BCG* signals, *x* is the lateral (left-to-right) axis, *y* is the longitudinal body (caudocranial) axis and *z* is the anteroposterior (ventrodorsal) axis; for *SCG* signals, the *z*-axis points in the opposite direction (dorsoventral) and the *x*-axis right-to-left.

### 2.3. Kinocardiography Data Analysis

Participant height and weight were used to assess their inertial parameters [12]. Linear accelerations ($\vec{a}$) first underwent single-time integration to provide velocity ($\vec{v}$). From this, the linear and rotational kinetic energy transmitted by cardiac contraction to the body were computed using the following equations on the ensemble averaged signals:

(1) $$K_{Lin}=\frac{1}{2}m(v_{x}^{2}+\;v_{y}^{2}+\;v_{z}^{2})$$

(2) $$K_{Rot}=\frac{1}{2}\left(I_{xx}\omega_{x}^{2}+I_{yy}\omega_{y}^{2}+\;I_{zz}\omega_{z}^{2}\right)$$

where m is the body mass of the subject; $K_{Lin}$ is the linear kinetic energy; $v_{x}$,$\;v_{y}$ and$\;v_{z}$ are the orthogonal components of the velocity vector $\vec{v}$; $K_{Rot}$ is the rotational kinetic energy; $I_{xx}$,$\;I_{yy}\;\mathrm{and}\;I_{zz}$ are the orthogonal components of the moment of inertia; and $\omega_{x}$,$\;\omega_{y},$ and$\;\omega_{z}$ are the orthogonal components of the angular velocity $\vec{\omega }$ measured from the gyroscopes. 

R waves were automatically detected on the ECG channel and served as fiducial points to delimit the cardiac beats through the record. The detection of R waves on the beat-by-beat ECG signal was checked visually on each signal and manually corrected when needed.

For each beat *j* of a record, the time integral (*iK*) of *K* over the entire cardiac cycle (CC) was computed as follows:

(3) $$SCG\;iK_{Lin}^{j}=\int_{CC}^{}SCG\;K_{Lin}\left(t\right)\;dt$$

(4) $$SCG\;iK_{Rot}^{j}=\int_{CC}^{}SCG\;K_{Rot}\left(t\right)\;dt$$

(5) $$BCG\;iK_{Lin}^{j}=\int_{CC}^{}BCG\;K_{Lin}\left(t\right)\;dt$$

(6) $$BCG\;iK_{Rot}^{j}=\int_{CC}^{}BCG\;K_{Rot}\left(t\right)\;dt$$

where CC was defined as starting with the P wave of the beat *j* and ending with the P wave of the beat *j* + 1, and delimited based on an ensemble averaged ECG computed on the whole record.

An RR interval time series analysis and classification procedure was used to exclude premature-ventricular contractions and non-sinus rhythm disturbances. Additionally, we applied an outlier detection on beats that would generate abnormally large energies, possibly due to involuntary movement of the subject, such as coughing, deglutition, or movements of the extremities. When the integral of *SCG* $iK_{}^{j}$ or *BCG* $iK_{}^{j}$ of a heartbeat was higher than 5 times the median of the respective kinetic energy of the 5 previous beats, the heartbeat was excluded from the computation. The following scalar metrics were then computed:

(7) $$SCG\;iK_{Lin}=\;\frac{\sum_{1}^{n}SCG\;iK_{Lin}^{j}}{n\;}$$

(8) $$SCG\;iK_{Rot}=\;\frac{\sum_{1}^{n}SCG\;iK_{Rot}^{j}}{n\;}$$

(9) $$BCG\;iK_{Lin}=\;\frac{\sum_{1}^{n}BCG\;iK_{Lin}^{j}}{n\;}$$

(10) $$BCG\;iK_{Rot}=\;\frac{\sum_{1}^{n}BCG\;iK_{Rot}^{j}}{n\;}$$

where *n* is the number of heartbeats in a record, $iK_{Lin}^{j}$ is the integrated linear energy of the *j*th heartbeat and $\;iK_{Rot}^{j}$ is the integrated rotational energy of the *j*th heartbeat.

### 2.4. Statistics

All data analyses were performed offline using a proprietary software toolbox developed by our team under MATLAB (MATHWORKS Inc.^®^, Natick, MA, USA).

#### 2.4.1. TRT Reliability Analysis

We used intraclass correlation coefficients (ICC) to assess *iK* KCG TRT reliability. Multiple variants of ICC exist, each with different advantages and limitations, first presented by Shrout et al. [25] and further developed by McGraw et al. [26]. The specific form used here is a two-way mixed model without interaction (Equation (11)). This model measures the absolute agreement of measurements made under the fixed level of the measurement factor.

(11) $$ICC_{c}=\frac{MS_{p}-\;MS_{e}}{MS_{p}+\left(k-1\right)MS_{e}+\frac{k}{n}\left(MS_{c}-\;MS_{e}\right)}$$

where *MS_p_* is the between-sessions (A, B and C) mean square representing the variability between sessions, *MS_e_* is a residual mean square traditionally referred to as mean square error of measurements, $MS_{c}$ is the between-participants mean square representing the variability between participants, *n* is the number of participants and *k* is the number of measurements. More details can be found in [26].

We assessed KCG parameter reliability as follows: (1) For each KCG parameter and HR, intrasession TRT reliability was calculated as the $ICC_{c}$ between time point A and time point B; see Figure 1. Intersession TRT reliability was computed as the $ICC_{c}$ between time point A and time point C. These analyses were performed for each position: Sup and LD individually.

In other studies [21], repeatability is also expressed as the coefficient of variation (*CV*) as:

(12) $$CV=\frac{\sigma }{\mu }$$

where μ is the mean and σ is the standard deviation of KCG parameters and HR between recordings of time point A and time point B. *CV* is a descriptive statistic and measures the variability of the data independently of the unit of measurements [27]. *CV* value has an inverse relation with repeatability, with a lower *CV* value representing higher repeatability.

#### 2.4.2. Influence of Position

KCG parameters and HR of time point A were compared between positions by a paired t-test for data with a normal distribution, or a Wilcoxon signed rank test for skewed data. A Lilliefors test was used to test whether the difference between the compared sample populations was normally distributed. As two-time points were compared, a *p*-value less than 0.05 was considered to compute 95% confidence intervals.

## 3. Results

**Figure 2:**
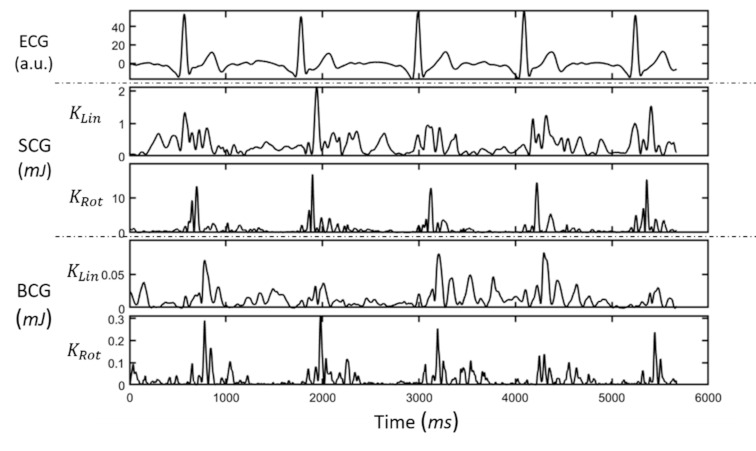
Waveforms of (from top to bottom) electrocardiogram (ECG), seismocardiography (*SCG*) linear kinetic energy, *SCG* rotational kinetic energy, ballistocardiography (*BCG*) linear kinetic energy and *BCG* rotational kinetic energy for a representative subject at baseline.

Figure 2 illustrates linear and rotational kinetic energy for *SCG* and *BCG* for a representative subject during baseline. Additionally, raw *BCG* and *SCG* linear accelerations and angular rates for a representative subject can be found in the Supplementary Materials (Figure S1). KCG parameters, HR and blood pressure (mean ± standard deviation) for the two positions (Sup and LD) during the first measure (A), together with short- and long-term TRT (B and C, respectively), are presented in Table 1.

### 3.1. Test–Retest Reliability

**Figure 3:**
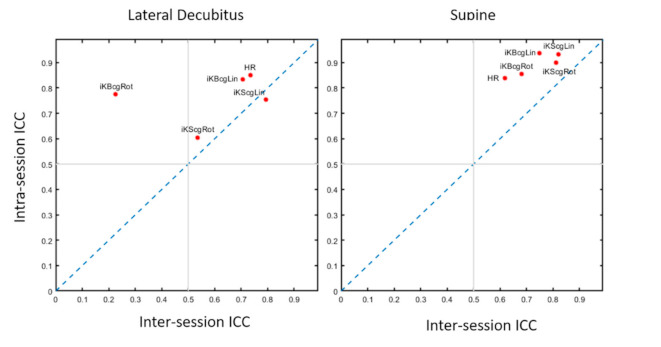
This scatter plot depicts the modal $ICC_{c}$ values appearing in both intersession (*x*-axis) and intrasession (*y*-axis) for each KCG *iK* parameter and HR. Two thin black lines are drawn to display the critical value ($ICC_{c}$ = 0.5) for both intra- and intersession test–retest reliability, and the blue dash line characterizes the positions with equal intra- and intersession reliability.

For each KCG parameter and HR, short- and long-term TRT reliability were generated as *CV* and $ICC_{c}$ (Table 2). Long-term TRT reliability of blood pressure is also presented. For all positions, all KCG parameters and HR exhibited moderate to high TRT reliability within sessions (modal $ICC_{c}$: 0.6–0.9). In supine, *SCG iK* metrics showed a higher TRT reliability both within and between sessions (modal $ICC_{c}$: 0.7–0.9) compared to LD (modal $ICC_{c}$: 0.2–0.8). KCG parameters and HR showed a higher TRT reliability within (modal $ICC_{c}$: 0.7–0.9) than between (modal $ICC_{c}$: 0.2–0.8) sessions. The highest intrasession TRT reliability was found for linear parameters *BCG* $iK_{Lin}^{}$ and *SCG* $iK_{Lin}^{}$ in supine (modal $ICC_{c},\;$respectively: 0.94 and 0.93) and *BCG* $iK_{Lin}^{}$ and *SCG* $iK_{Lin}^{}$ in LD (modal $ICC_{c,\;}$respectively: 0.84 and 0.80). The lowest intrasession TRT reliability was found for rotational parameters *SCG* $iK_{Rot}^{}$ in LD (modal $ICC_{c}$: 0.61) and *BCG*$\;iK_{Rot}^{}$ in LD (modal $ICC_{c}$: 0.77). The highest intersession TRT reliability was found for *SCG* $iK_{Lin}^{}$ and *SCG* $iK_{Rot}^{}$ in supine (modal $ICC_{c}$: 0.82 and 0.82, respectively) and *SCG* $iK_{Lin}^{}$ in LD (modal $ICC_{c}$: 0.79). The lowest intersession TRT reliability was found for *BCG* $iK_{Lin}^{}$ and *BCG* $iK_{Rot}^{}$ in supine (modal $ICC_{c},\;$respectively: 0.76 and 0.70) and *BCG* $iK_{Rot}^{}$ in LD (modal $ICC_{c}$: 0.22). Detailed $ICC_{c}$ with a 90% Confidence Interval (CI) can be found in Table 2 for each position, within and between sessions. Figure 3 shows the intra- and intersession $ICC_{c}$ modal values for each position. The TRT reliabilities in men and women were in the same range.

Intrasession *CV* values of KCG parameters were all below 4.5% for supine, and below 5.5% for LD.

### 3.2. Influence of Position

**Figure 4:**
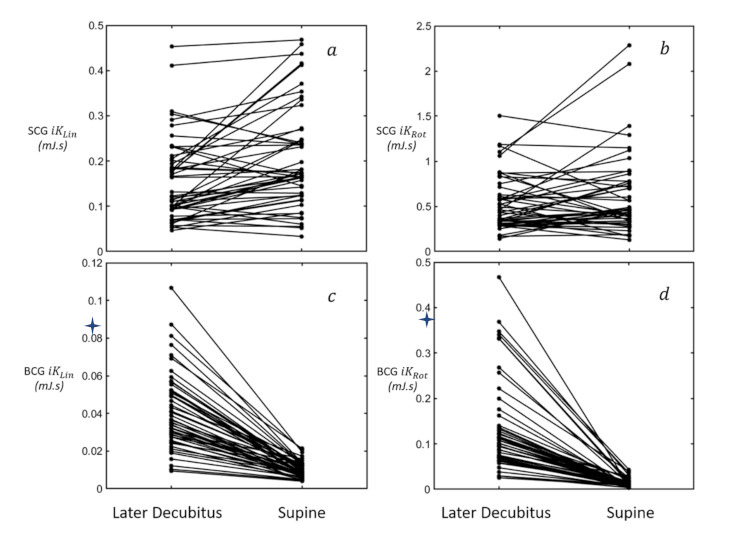
KCG metrics measured in two positions: LD and supine at collection point A. (**a**) *SCG* $iK_{Lin}$>; (**b**) *SCG* $iK_{Rot}$; (**c**) *BCG* $iK_{Lin}$; (**d**) *BCG* $iK_{Rot}$.*: *p* < 0.0001.

Paired *t*-tests were used to compare LD and supine positions for these parameters, to assess how hemodynamic changes caused by the transition from supine to LD impact KCG metrics. The *BCG* $iK_{Lin}$ and *BCG* $iK_{Rot}$ decreased from LD to supine (*p* < 0.005). The *SCG* $iK_{Lin}$ and *SCG* $iK_{Rot}$ were stable from LD to supine (Figure 4).

## 4. Discussion

### 4.1. Main Findings

The present work evaluated the reliability and reproducibility of automatically computed KCG metrics in supine and left lateral decubitus positions, yielding three main results. First, we found high short-term TRT reliability for KCG metrics derived from *SCG* and *BCG* recordings acquired while the volunteers lay in supine position. Second, our analyses revealed that the TRT reliability of KCG metrics was affected by the volunteer position, and unlike in supine position, the measures acquired in lateral decubitus led to moderate TRT reliability, as further discussed in Section 4.2. Finally, we found moderate long-term (3 to 6 h) TRT reliability for KCG metrics as expected and confirmed by variation of blood pressure taken at these two-time points. It should be noted that the metrics were computed automatically, without any action from an operator to select particular heartbeats, for instance. The present results are thus a good test case before using this technique for cardiac telemonitoring, where automation of the postprocessing is highly desired.

### 4.2. Test–Retest Reliability and Reproducibility

Test–retest in amplitude measures from ballistocardiographic signals acquired on an air bed has been studied with contradictory results, some studies showing large reproducibility [18], while others showing significant but lower reproducibility [19]. With the development of new technologies such as ElectroMechanical Film (EMFi) sensors, high-precision scales, MEMS accelerometers and gyroscopes, such repeatability studies were performed on the *BCG* amplitude with consistent satisfying results in sitting position [20] or in standing position [28]. For both studies, the tests were performed in a single position. Short- and long-term (1 h and 1 day, respectively) systolic time interval repeatability derived from *SCG* signals was also recently studied by Vahid Zakeri et al. [21]. They found a *CV* of less than 2 and 3% for the short- and long-term repeatability, respectively. To our knowledge, repeatability of the *SCG* amplitude signal on a single axis or a combination of axes has never been studied. Our study demonstrates that KCG metrics derived from *SCG* (*SCG* $iK_{Lin}^{}$, *SCG* $iK_{Rot}^{}$), and those derived from *BCG* (*BCG* $iK_{Lin}^{},$ and *BCG* $iK_{Rot}^{}$), computed on signals acquired 10 to 15 min apart in supine position are repeatable with an $ICC_{c}$ on average greater than 0.85 and *CV* values lower than 5.5%. The gender of the volunteers did not affect these results.

In a previous study, *BCG* $iK_{Lin}^{}$ was shown to be correlated with stroke volume (SV) acquired by echocardiography [12]. Others have shown that SV measured at submaximal exercise by doppler echocardiography has an $ICC_{c}$ of 0.79 and a *CV* of 12% [29]. The *BCG* $iK_{Lin}^{}$ repeatability scores ($ICC_{c}$ of 0.84–0.94 and *CV* of 2.85–4.35%), therefore, seem to be in line with these results. As expected, the comparison of measurement performed in the morning and 3 to 6 h later gave moderate $ICC_{c}$ values from 0.2 to 0.8; this is likely due to intraday physiological variations in cardiovascular parameters, as confirmed by an observed low $ICC_{c}$ value for variation in blood pressure (Table 2). This suggests that for a conventional longitudinal monitoring of patients at home, each KCG measure should be performed at the same time of the day, preferably in the morning when the activity performed immediately before may be the most similar.

### 4.3. Postural Changes Effects

Postural changes are detectable in the amplitude of *BCG* [30] and *SCG* [31] signals. In particular, according to Taebi et al. [32], the transition from supine to left hand lateral decubitus leads to an increase in the amplitude of *BCG* y axis signal. This is confirmed in our study by an increase in *BCG* based *iK* energies, both linear and rotational, when compared to the acquisition in supine position. This comes as no surprise as stroke volume is higher in LD than in supine due to the increase in right ventricular diastolic filing [33]. However, when acquisitions were performed in LD, *BCG iK* metrics showed higher variability and a low-to-moderate TRT value. This may be due to the weaker mechanical coupling of the *BCG* sensor in LD when not pressed on the bed, as is the case in supine position.

### 4.4. Future Directions and Perspectives

This study highlights that KCG parameters derived from *BCG* and *SCG* are more repeatable when acquired in supine than in left lateral decubitus position. In addition, the high short-term repeatability of KCG metrics after de-instrumenting and re-instrumenting the volunteers shows that this technique is robust to small errors in sensors placement.

The sensitivity of KCG to throughout-the-day variations of hemodynamic parameters found in this study could also be used. Indeed, it is known that BP has a diurnal pattern, and it has been suggested that circadian patterns and night-time blood pressure values may be more highly correlated with indices of end-organ damage than resting blood pressure values [34]. While this phenomenon was extensively studied due to the availability of ambulatory blood pressure recordings [35], the sensitivity of KCG to other cardiovascular parameters, more correlated with SV, may open the possibility to study new diurnal patterns predicting cardiovascular failure.

Phasic respiratory variation in amplitude of the *BCG* waves, modest in normal subjects, is accentuated in heart disease [36]. Abnormalities in the force of left ventricular ejection are masked during the inspiration phase and may be revealed only in the expiratory phase of breathing, when right ventricular ejection is reduced [36]. Given the importance of metrics computed in a single respiratory phase, the TRT repeatability of each KCG metric over different respiratory phases could also be studied in future studies. Additionally, this study only covered intraday repeatability. A following study should focus on an interday repeatability analysis where the KCG measure is performed at the same time of the day, preferably in the morning when the activity performed immediately before may be the most similar. *SCG* and *BCG* are both sensitive to movement-induced noises, from leg movements to swallowing. Solutions are proposed in the literature to remove motion artefacts and are based on digital filtering [37,38] or a second sensor placed on the scapula serving as a reference [39]. However, in all these situations, the scope is to detect feature points on the *SCG*. These solutions do not focus on a possible distortion of the signals in amplitude. As KCG parameters rely on *SCG*/*BCG* amplitudes, these solutions are less relevant. In this work, the motion artefacts issue was tackled in two ways: (1) by instructing our volunteers to stay as still as possible during the protocols; (2) through a beat-by-beat elimination based on a moving median. This method is based on the hypothesis that only a minority of heartbeat signals are distorted by motion artefacts. Therefore, these solutions may have given satisfactory results in the present study because they were performed in a controlled environment and under the supervision of an experimented operator. For at home use, this solution still heavily relies on the patient’s willingness or capacity to remain as still as possible, and alternative techniques to tackle this issue should be investigated in future studies.

### 4.5. Limitations

The present work also presents some limitations worth noting. The *SCG* sensor was attached to the body of each participant with ECG electrodes and was not further secured with a belt. This may induce a change in the mechanical coupling of the sensors to the body especially in decubitus lateral position, further explaining the moderate TRT reliability found in this study for this position.

Additionally, these validations were performed on a relatively young group of participants. Given that the order of positioning from LD to supine was not randomized, the effect of hemodynamic parameter stabilization through the procedure may have impacted the TRT reliability of the measures. However, the potential effect of positioning order was minimized, as participants were placed in each position for 5 min before the beginning of acquisition.

## 5. Conclusions

This study demonstrates that KCG and its kinetic energy scalar parameters, based on 12-DOF signals, computed automatically on records acquired in supine position 10 to 15 min apart are repeatable. As expected, comparing measures performed in the morning and 3 to 6 h later gives moderate $ICC_{c}$ values from 0.7 to 0.8. The body position has an impact on repeatability, as the KCG metrics computed in supine position showed better repeatability than those computed in left lateral decubitus position. In light of this, the recording of body vibrations produced by myocardial contraction and blood flow using MEMS is a technique showing high repeatability that could, therefore, be used to longitudinally and non-invasively telemonitor cardiac inotropic activity.

## Figures and Tables

**Table 1:** Kinocardiography (KCG) parameters, heart rate (HR), systolic blood pressure (BPsys) and diastolic blood pressure (BPdia) (mean ± standard deviation) for the two configurations, supine (Sup) and left lateral decubitus (LD), during the first measure (A), together with short- (B) and long-term (C) test–retest (TRT).

Measurements		$BCG\;iK_{Lin}^{}\;$ *(µJ·s)*	$BCG\;iK_{Rot}^{}\;$ *(µJ·s)*	*SCG* $iK_{Lin}^{}$ *(µJ·s)*	*SCG* $iK_{Rot}^{}$ *(µJ·s)*	HR (bpm)	BPsys (mmHg)	BPdia (mmHg)
Beginning (A)	Sup	9.7 ± 3.5	11.9 ± 6.3	195.3 ± 99.4	564.6 ± 316.8	67.1 ± 9.1	128.3 ± 11.9	77.6 ± 9.2
	LD	37.6 ± 17.5	108.9 ± 73.9	131.7 ± 64.0	489.0 ± 264.5	68.2 ± 9.8	128.4 ± 12.2	77.0 ± 9.6
10 to 15 min later (B)	Sup	10.4 ± 4.2	13.8 ± 9.0	196.5 ± 110.6	609.5 ± 390.0	65.0 ± 8.7	128.5 ± 12.1	77.1 ± 9.2
	LD	34.0 ± 14.1	89.2 ± 44.9	102.1 ± 42.1	383.0 ± 159.3	64.7 ± 9.1	128.0 ± 11.8	77.2 ± 9.4
3 to 6 h later (C)	Sup	11.8 ± 4.4	18.3 ± 9.9	206.3 ± 88.6	827.1 ± 549.1	66.0 ± 10.5	130.0 ± 9.5	80.6 ± 8.3
	LD	37.8 ± 19.4	103.0 ± 63.2	134.9 ± 74.3	479.7 ± 289.5	69.0 ± 9.9	129.5 ± 9.8	80.2 ± 8.6

**Table 2:** Short- and long-term test–retest (TRT) described as coefficient of variation (*CV* (%)) and intraclass correlations ($ICC_{c}$ (mean [CI 90%])) for KCG parameters, heart rate (HR), systolic blood pressure (BPsys) and diastolic blood pressure (BPdia) for the 2 configurations: supine-1 (Sup) and left lateral decubitus (LD).

	Measurements	$BCG\;iK_{Lin}^{}\;$	$BCG\;iK_{Rot}^{}\;$	$SCG\;iK_{Lin}^{}\;$	$SCG\;iK_{Rot}^{}\;$	HR	BPsys	BPdia
Short-term TRT (A vs. B)	*CV* Sup (%)	2.85	3.48	4.54	3.98	1.77	NA	NA
	*CV* LD (%)	4.35	5.07	1.82	5.34	1.1	NA	NA
	*ICC_c_* Sup ([90% CI])	0.94 [0.91;0.96]	0.86 [0.79;0.91]	0.93 [0.90;0.96]	0.90 [0.85;0.94]	0.84 [0.69;0.91]	NA	NA
	*ICC_c_* LD ([90% CI])	0.84 [0.76;0.89]	0.77 [0.66;0.85]	0.80 [0.70;0.87]	0.61 [0.45;0.73]	0.85 [0.52;0.93]	NA	NA
Long-term TRT (A vs. C)	*ICC_c_* Sup ([90% CI])	0.76 [0.65;0.84]	0.70 [0.56;0.80]	0.82 [0.73;0.88]	0.82 [0.73;0.88]	0.62 [0.46;0.74]	0.31 [0.09;0.50]	0.50 [0.31;0.65]
	*ICC_c_* LD ([90% CI])	0.70 [0.57;0.80]	0.22 [0.00;0.42]	0.79 [0.69;0.86]	0.53 [0.35;0.68]	0.74 [0.61;0.82]	0.40 [0.19;0.57]	0.44 [0.24;0.60]

## Data Availability

The datasets used and/or analysed during the current study are available from the corresponding author on reasonable request.

## Supplementary Materials

The following are available online at https://www.mdpi.com/1424-8220/21/3/815/s1. Figure S1: Raw data of a simultaneously acquired electrocardiogram (ECG); 6D seismocardiogram (*SCG*); 6D ballistocardiogram (*BCG*), from one representative subject in supine position.
