# Supplementary figures and images for: Kinocardiography Derived from Ballistocardiography and Seismocardiography Shows High Repeatability in Healthy Subjects
Source: Sensors (Basel). 2021 Jan 26;21(3):815. doi: 10.3390/s21030815 (PMC7865512; doi:10.3390/s21030815)

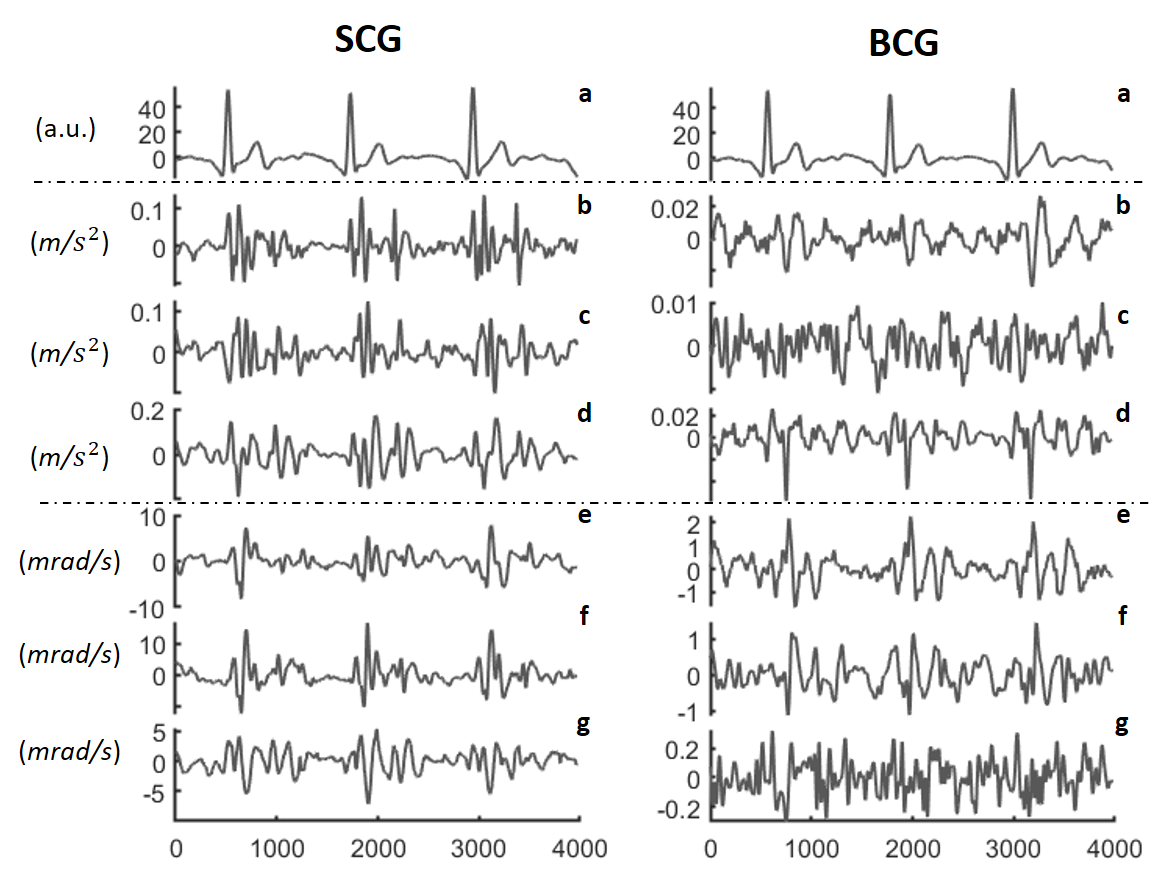

Supplement: Supplementary file 1 [file sensors-21-00815-s001.zip › sensors-1033781-supplementary.bmp]
